# Supplementary material for: Phosphonopeptides Revisited, in an Era of Increasing Antimicrobial Resistance
Source: Molecules. 2020 Mar 23;25(6):1445. doi: 10.3390/molecules25061445 (PMC7144462; doi:10.3390/molecules25061445)
Supplement: Supplementary file 1 [file molecules-25-01445-s001.pdf]

Phosphonopeptides Revisited, in an Era of Increasing Antimicrobial Resistance

Emma C.L. Marrs [1,2], Linda Varadi [3], Alexandre F. Bedernjak [3], Kathryn M. Day [1], Mark Gray [3], Amanda L. Jones [2], Stephen P. Cummings [2], Rosaleen J. Anderson [3,†] and John D. Perry [1,*]

1 Department of Microbiology, Freeman Hospital, Newcastle upon Tyne NE7 7DN, UK
2 Department of Applied Sciences, Northumbria University, Newcastle upon Tyne NE1 8ST, UK
3 Sunderland Pharmacy School, University of Sunderland, Sunderland, SR1 3SD, UK
* Correspondence: john.perry5@nhs.net
† Deceased.

S1 Supplementary information:

1. General

NMR spectra were obtained on a Bruker Ultrashield 300 spectrometer (at 300 MHz for ^{1}H and at 75 MHz for ^{13}C spectra). The chemical shifts are shown in ppm downfield from tetramethylsilane, using residual chloroform (δ=7.26 in ^{1}H NMR) or the middle peak of $CDCl_3$ carbon triplet (δ=77.23 in ^{13}C NMR) as an internal standard. Melting points were obtained using a Reichart-Kofler hot-stage microscope apparatus and are uncorrected. Infrared spectra were recorded using a PerkinElmer Spectrum BX FT-IR instrument. Low resolution mass spectra were recorded on a Bruker Esquire 3000plus analyser using electrospray source in positive ion mode. High resolution mass spectra were obtained on a LTQ Orbitrap XL instrument in nanospray ionization mode. Elemental analyses were carried out using an Exeter Analytical CE-440 Elemental Analyzer. All commercially available reagents and solvents were obtained from Sigma-Aldrich, Alfa-Aesar, Fisher Scientific and Fluka and were used without any further purification. Thin layer chromatography was carried out on Merck silica gel plates (60F-254).

2. Preparation of L-alanyl-L-alanyl-L-fosfalin

Synthetic pathway for the preparation of L-alanyl-L-alanyl-L-fosfalin:

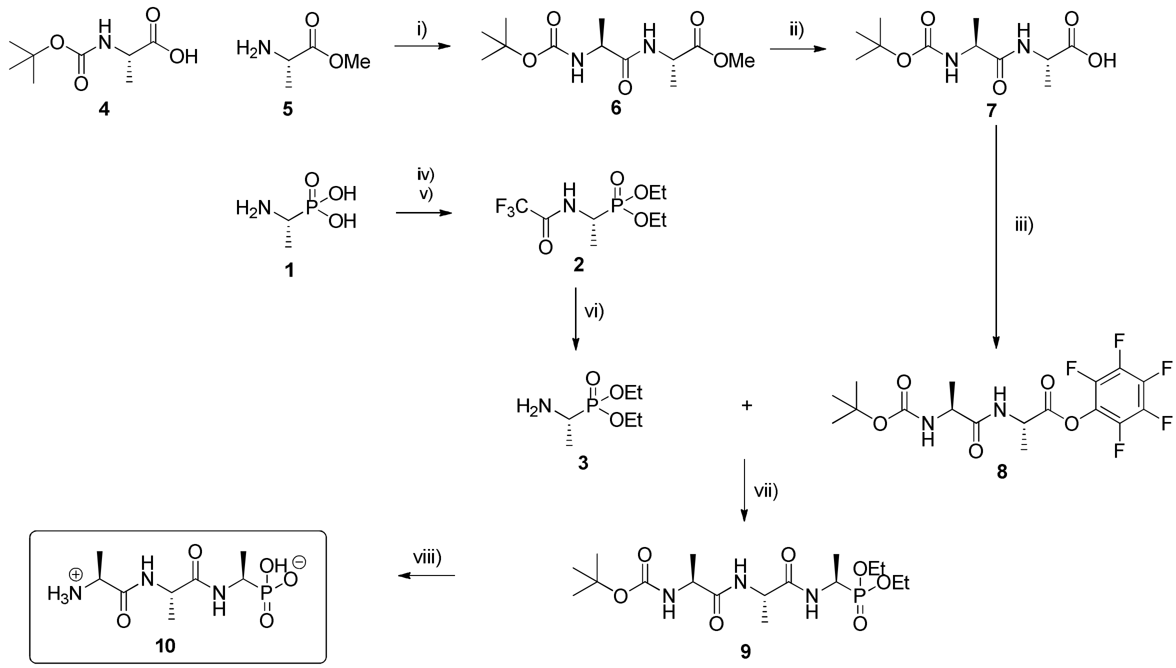

Scheme 1 Synthetic route for the preparation of Ala-Ala-Fos **10**

Reagents and conditions: i) DCC, HOBt, DCM, EtN(iPr)$_2$, 0 °C to r.t.; ii) NaOH, MeOH; iii) DCC, pentafluorophenol, EtOAc, 0 °C; iv) CF_3COOH, $(CF_3CO)_2O$, 55 °C, 25 min; v) HC(OEt)$_3$, 110 °C, 2 h; vi) $NaBH_4$, EtOH; vii) DIPEA, DMF; viii) HBr in AcOH, then propylene oxide

2.1 (*R*)-Diethyl [1-(trifluoroacetamido)ethyl]phosphonate 2 [1, 2]

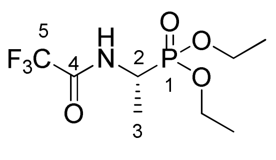

L-1-Aminoethyl phosphonic acid **1** (2.00 g, 16.0 mmol) was dissolved in a mixture of trifluoroacetic acid (2 mL) and trifluoroacetic anhydride (9.8 mL). The solution was stirred at 55 °C for 30 minutes, followed by the dropwise addition of triethylorthoformate

(60 mL). The reaction mixture was then heated at 110 °C for 2 h. After evaporation of the volatile components, purification by column chromatography (90% DCM, 10% MeOH) gave diethyl [1-(trifluoroacetamido)ethylphosphonate **2** as an oil (3.88 g, 14.0 mmol, 87%); [Found: C, 34.07; H, 5.25; N, 5.09. $C_8H_{15}F_3NO_4P$ requires C, 34.67; H, 5.45; N, 5.05 %]; ν_{max}/cm^{-1} 3205 (NH), 1717 (C=O), 1559 (amide II), 1206, 1181, 1151 (P=O), 1048, 1018, 971 (C-F); ^{1}H NMR (300 MHz, $CDCl_3$) δ_H 1.25-1.36 (6H, overlapping 2xt, 2 x CH_3), 1.44 (3H, dd, *J*=7.3 and 16.7 Hz, CH_3-3), 4.07-4.19 (4H, overlapping 2xq, 2 x CH_2), 4.37-4.53 (1H, m, CH-2), 7.63 (1H, d, *J*=7.3 Hz, NH); ^{13}C NMR (75 MHz, $CDCl_3$) δ_C 14.9 (CH_3, C-3), 16.2 (CH_3, d, *J*=2.25 Hz, OCH_2CH_3), 16.3 (CH_3, d, *J*=2.25 Hz, OCH_2CH_3), 41.7 (CH, d, *J*=158.9 Hz, C-2), 63.0 (CH_2, d, *J*=6.75 Hz, OCH_2), 63.2 (CH_2, d, *J*=6.75 Hz, OCH_2), 117.7 (CF_3, q, *J*=285.9 Hz), 156.5 (quat., m, C=O); ^{31}P NMR (121.5 MHz, $CDCl_3$) δ_P 23.0 (m); HRMS (NSI) calcd for $(C_8H_{16}F_3NO_4P)^+$ 278.0764, found 278.0762.

2.2 (*R*)-Diethyl 1-aminoethylphosphonate 3 [3,4]

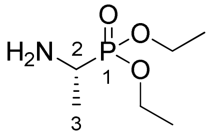

Diethyl [1-(trifluoroacetamido)ethylphosphonate **2** (4.39 g, 16.0 mmol) was dissolved in ethanol (200 mL) and sodium borohydride (3.60 g, 96.0 mmol) was added in small portions. The reaction mixture was stirred at room temperature for 1 hour, followed by reflux for 30 minutes. The solvent was removed under reduced pressure. The residue was treated with saturated $NaHCO_3$ (150 mL) and extracted with DCM (5x 50 mL). The organic layer was dried over Na_2SO_4 and the solvent was removed under vacuum. The obtained oil was purified by column chromatography (95% DCM, 5% MeOH) to give

phosphonate **3** as a colourless oil (1.98 g, 11.0 mmol, 68%); ν_{max}/cm^{-1} (liquid film) 3434 (br NH), 2980, 2905, 1217 (P=O), 1019; ^{1}H NMR (300 MHz, $CDCl_3$) δ_H 1.29-1.35 (9H, s, 3 x CH_3), 3.03-3.13 (1H, m, CH-2), 4.07-4.18 (4H, overlapping 2xq, 2 x CH_2); ^{13}C NMR (75 MHz, $CDCl_3$) δ_C 16.5 (CH_3), 16.6 (CH_3), 17.2 (CH_3), 44.2 (CH, d, J=148.5 Hz, C-2), 62.0-62.2 (CH_2, m, 2 x OCH_2); ^{31}P NMR (121.5 MHz, $CDCl_3$) δ_P 29.62 (m); HRMS (NSI) calcd for $(C_6H_{17}NO_3P)^+$ 182.0941, found 182.0936.

2.3 ^{t}Boc-L-alanyl-L-alanine methyl ester 6 [5]

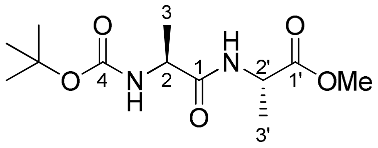

^{t}Boc-L-alanine **4** (4.62 g, 24.4 mmol) and alanine methyl ester **5** (3.09 g, 22.1 mmol) were suspended in dichloromethane (150 mL) and HOBt (3.88 g, 28.7 mmol) was added. The mixture was cooled in an ice bath and, after 15 minutes, *N,N*-diisopropylethylamine (4.23 g, 5.7 mL, 33.2 mmol) was slowly added. The resulting clear solution was stirred for 10 minutes at 0 °C. *N,N*-Dicyclohexylcarbodiimide (5.93 g, 28.7 mmol) was added portionwise and the reaction mixture was stirred for 2 h at 0 °C. Stirring was then continued at room temperature for an additional 72 h. Upon completion, the urea precipitate that had formed was removed by filtration. The solvent was removed under reduced pressure and the residue was purified by column chromatography (60% petroleum ether, 40% ethyl acetate) to give the product **6** as a white solid (4.89 g, 17.9 mmol, 81%); mp 107-109 °C (lit. mp 112-113 °C); [Found: C, 52.67; H, 8.12; N, 10.19. $C_{12}H_{22}N_2O_5$ requires C, 52.54; H, 8.08; N, 10.21 %]; ν_{max}/cm^{-1} 3307 (NH), 2979 (NH), 1738 (C=O, ester), 1676 (C=O), 1653 (C=O), 1550 (amide II), 1521 (amide II); ^{1}H NMR (300 MHz, $CDCl_3$) δ_H 1.31 (3H, d, *J*=6.9 Hz, CH_3), 1.40 (3H, d, *J*=7.2 Hz, CH_3), 1.45

(9H, s, $C(CH_3)_3$), 3.74 (3H, s, OCH_3), 4.13 (1H, quint., *J*=7.2 Hz, CH), 4.57 (1H, quint., *J*=7.2 Hz, CH), 5.01 (1H, d, *J*=6.6 Hz, NH), 6.65 (1H, br, NH); ^{13}C NMR (75 MHz, $CDCl_3$) δ_C 18.2 (CH_3), 18.3 (CH_3), 28.3 ($C(CH_3)_3$), 48.1 (OCH_3), 50.3 (CH), 52.4 (CH), 81.3 (quat., $C(CH_3)_3$), 172.2 (2 x quat., 2 x C=O, C-4 and 1), 173.1 (quat., C-1'); MS (ESI) *m/z* 297.3 (MNa^+).

2.4 ^{t}Boc-L-alanyl-L-alanine 7 [6]

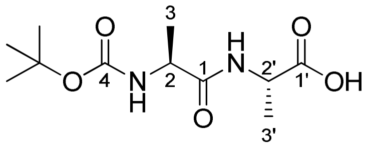

^{t}Boc-L-alanyl-L-alanine methyl ester **6** (4.03 g, 14.7 mmol) was dissolved in methanol (50 mL) and 1M NaOH (65 mL) was added. The reaction mixture was stirred at room temperature for 6 h. After evaporation of the volatiles, the residual water layer was extracted with DCM. The water layer was cooled and acidified with concentrated hydrochloric acid to pH 1-2. The mixture was diluted with water (100 mL) and maintained at 5 °C overnight. The white solid was isolated by filtration and washed with water to give the product **7** as a white crystalline solid (3.34 g, 12.8 mmol, 87%); mp 111-116 °C (lit. mp 98 °C (7)); ν_{max}/cm^{-1} 3428 (br, OH), 3347 (NH), 2976 (NH), 1664 (br, C=O), 1534 (amide II); ^{1}H NMR (300 MHz, DMSO-*d*$_6$) δ_H 1.17 (3H, d, *J*=7.2 Hz, CH_3), 1.27 (3H, d, *J*=7.2 Hz, CH_3), 1.37 (9H, s, $C(CH_3)_3$, 3.98 (1H, quint., *J*=7.2 Hz, CH), 4.20 (1H, quint., *J*=7.2 Hz), 6.83 (1H, d, *J*=7.5 Hz, NH), 7.96 (1H, d, *J*=7.5 Hz, NH); ^{13}C NMR (75 MHz, DMSO-*d*$_6$) δ_C 17.2 (CH_3), 18.0 (CH_3), 28.0 (CH_3, $C(CH_3)_3$), 47.2 (CH), 49.2 (CH), 77.9 (quat., $C(CH_3)_3$), 154.9 (quat., C=O), 172.3 (quat., C=O), 173.9 (quat., C=O); HRMS (NSI) calcd for $(C_{11}H_{19}N_2O_5)^-$ 259.1299, found 259.1294.

2.5 ^{t}Boc-L-alanyl-L-alanine pentafluorophenol ester 8

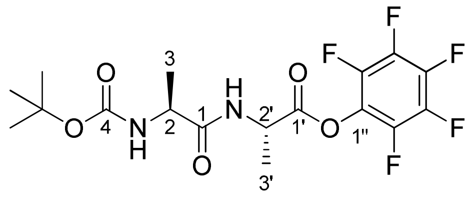

^{t}Boc-L-alanyl-L-alanine **7** (1.10 g, 4.2 mmol) and pentafluorophenol (0.85 g, 4.6 mmol) was dissolved in ethyl acetate (50 mL) and cooled in an ice bath. Dicyclohexylcarbodiimide (0.95 g, 4.6 mmol) was added and the reaction was stirred at 0 °C for a further 2 h. The precipitated urea was filtered off, the solvent was concentrated and the residue was re-dissolved in small amount of ethyl acetate (20 mL). Additional urea that has precipitated was removed by filtration. After removal of the ethyl acetate, the oil was triturated with petroleum ether to give **8** as a white crystalline solid (1.47 g, 4.1 mmol, 96%); mp 97-98 °C; ν_{max}/cm^{-1} 3334 (NH), 3311 (NH), 2995 (NH), 2941 (NH), 1787 (C=O), 1679 (C=O), 1656 (C=O), 1522 (amide II), 1515 (amide II); ^{1}H NMR (300 MHz, $CDCl_3$) δ_H 1.38 (3H, d, *J*=7.2 Hz, CH_3), 1.45 (9H, s, $C(CH_3)_3$), 1.61 (3H, d, *J*=7.2 Hz, CH_3), 4.21 (1H, quint., *J*=7.2 Hz, CH), 4.90 (1H, quint., *J*=7.2 Hz, CH), 4.97 (1H, br, NH), 6.80 (1H, br, NH). The crude product was used directly in the next reaction step.

2.6 ^{t}Boc-L-alanyl-L-alanyl-fosfalin diethyl ester 9 [7,8]

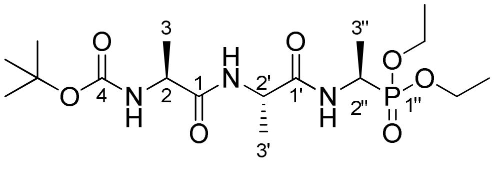

^{t}Boc-L-alanyl-L-alanine pentafluorophenol ester **8** (1.00 g, 2.35 mmol), diethyl 1-aminophosphonate **3** (0.42 g, 2.35 mmol) and diisopropylethylamine (0.30 g, 2.35 mmol) was dissolved in dry DMF (25 mL) and stirred overnight at room temperature. After completion the reaction was quenched with 1M HCl (50 mL) solution and extracted with

DCM (2 x 50 mL). The organic layer was washed with $NaHCO_3$ (30 mL) and water (30 mL). After drying over anhydrous sodium sulfate the solvent was removed and the crude product was purified by column chromatography (90% DCM, 10% MeOH) to give **9** as a white solid (0.95 g, 2.2 mmol, 95%); mp 168.4-172.6 °C; [Found: C, 47.77; H, 7.92; N, 9.51. $C_{17}H_{34}N_3O_7P$ requires C, 48.22; H, 8.09; N, 9.92 %]; ν_{max}/cm^{-1} 3311 (NH), 3276 (NH), 2973 (NH), 2357 (O=P-O), 1689 (C=O), 1672 (C=O), 1635 (C=O), 1519 (br, amide II), 1238 (P-O), 1167 (P-O); ^1H NMR (300 MHz, $CDCl_3$) δ_H 1.30-1.41 (15H, m, 5 x CH_3), 1.45 (9H, s, $C(CH_3)_3$), 4.10-4.16 (5H, m, 2 x CH_2 and CH), 4.39-4.50 (2H, m, 2 x CH), 4.97 (1H, br, NH), 6.53 (1H, d, *J*=8.7 Hz, NH), 6.66 (1H, d, *J*=7.5 Hz, NH); ^{13}C NMR (125 MHz, $CDCl_3$) δ_C 15.7 (CH_3, d, *J*=8.3 Hz, C-3”), 16.6 (CH_3, m, 2 x $OCH_2\mathit{CH_3}$), 16.7 (CH_3), 18.7 (CH_3), 28.5 (CH_3, $C(CH_3)_3$), 41.2 (CH, d, *J*=157.2 Hz, C-2”), 49.0 (CH), 49.1 (CH), 62.7 (CH_2, d, *J*=6.9 Hz, OCH_2), 62.8 (CH_2, d, *J*=6.7 Hz, OCH2), 80.2 (quat., $C(CH_3)_3$), 150.0 (quat., C=O), 171.9 (quat., C=O), 172.7 (quat., C=O); ^{31}P NMR (121.5 MHz, $CDCl_3$) δ_P 24.89 (m); HRMS (NSI) calcd for $(C_{17}H_{35}N_3O_7P)^+$ 424.2207, found 424.2204.

2.7 L-Alanyl-L-alanyl-L-fosfalin 10 [9]

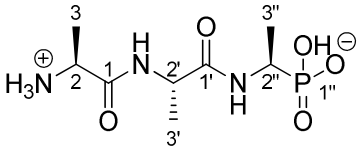

^{t}Boc-L-alanyl-L-alanyl-L-fosfalin diethyl ester **9** (2.24 g, 5.3 mmol) was dissolved in acetic acid (5 mL) and HBr in AcOH (33 w/w %) (30 mL) was added. The solution was stirred at room temperature for 24 h, and then quenched with diethyl ether (400 mL). After standing in the freezer (-15 °C) for 4 h, a brown oil formed and was separated by decanting off the diethyl ether. The crude product was washed with cold diethyl ether (5x

100 mL). The residue was taken up into methanol (10 mL) and the product **10** was obtained as a white solid (0.90 g, 2.6 mmol, 49%) on addition of large excess of propylene oxide (250 mL); mp 271.1-272.6 °C; ν_{max}/cm^{-1} 3329 (NH), 3256 (NH), 1640 (C=O), 1545, 1529, 1442 (P-C), 1152 (P=O), 1018 (P-OH), 924 (P-OH), 717 (P-C); ^{1}H NMR (300 MHz, D_2O) δ_H 1.27 (3H, dd, *J*=7.2 and 15.3 Hz, CH_3-3"), 1.38 (3H, d, *J*=7.2 Hz, CH_3), 1.53 (3H, d, *J*=7.2 Hz, CH_3), 3.94-4.11 (2H, m, 2 x CH), 4.29-4.36 (1H, q, *J*=7.2 Hz, CH); ^{13}C NMR (75 MHz, D_2O) δ_C 15.4 (CH_3, C-3"), 16.5 (2 x CH_3), 43.7 (CH, d, *J*=147.2 Hz, C-2"), 48.9 (CH), 50.0 (CH), 170.5 (quat., C=O), 173.6 (quat., C=O); MS (ESI) *m/z* 265.0 $(M-2H)^-$; HRMS (NSI) calcd for $(C_8H_{17}N_3O_5P)^-$ 266.0911, found 266.0905.

3. Preparation of β-chloro-L-alanyl-β-chloro-L-alanine 19

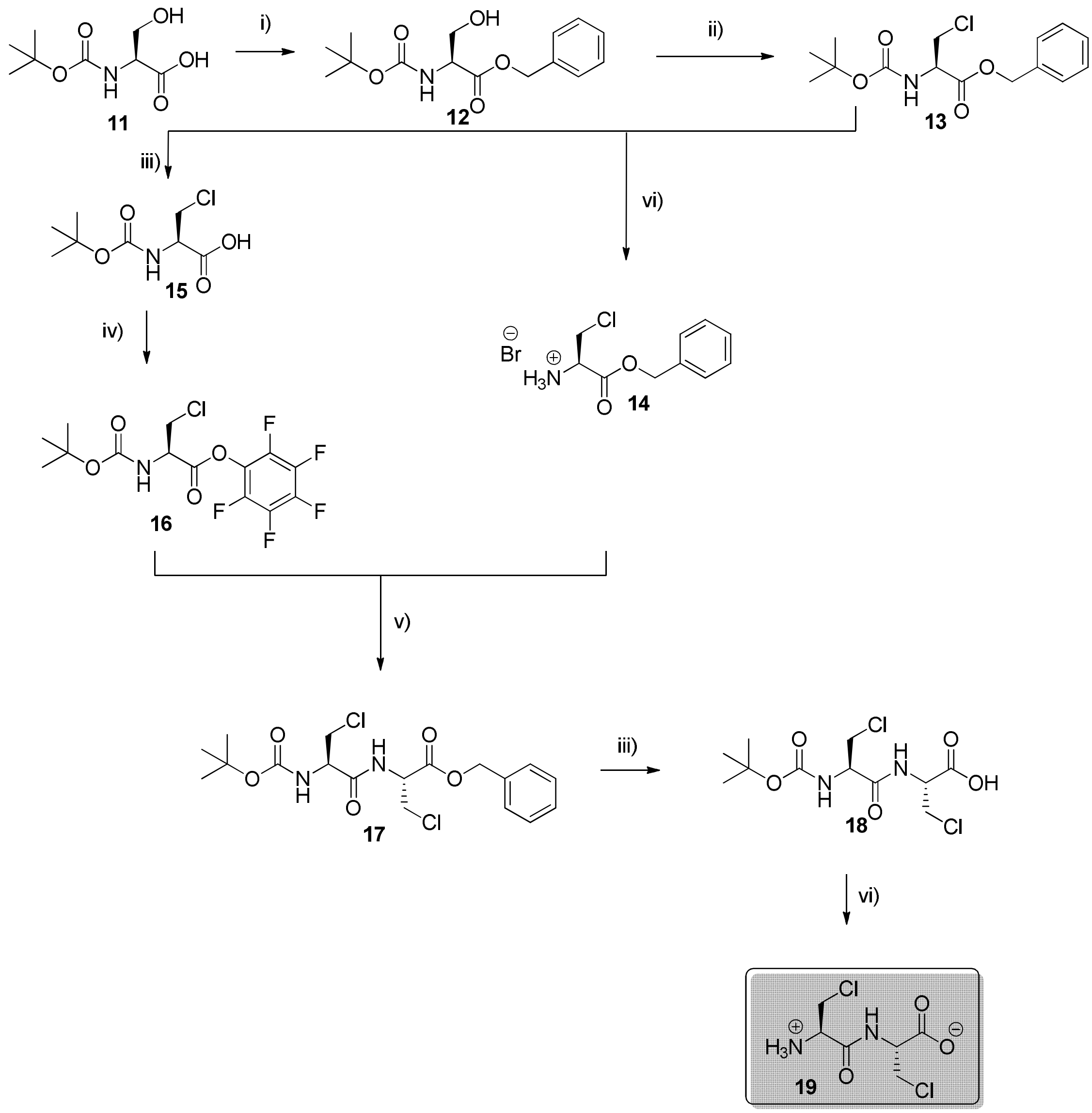

Scheme 2 Synthetic route for the preparation of β-chloroalanyl-β-chloroalanine **19**

Reagents and conditions: i) benzyl bromide, DBU, benzene; ii) Cl_3CCN, PPh_3, DCM, N_2, r.t.; iii) H_2, 10% Pd/C, MeOH; iv) PFP, DCC, EtOAc; v) DCC, DCM, DMF; vi) HBr, AcOH, then propylene oxide

3.1 ^{t}Boc-L-serine benzyl ester 12 [10]

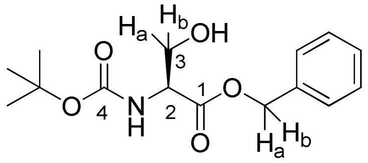

^{t}Boc-Serine **11** (16.60 g, 81.0 mmol) was dissolved in benzene (250 mL) and DBU (18.90 g, 21.6 mL, 124.0 mmol) was added. To the stirred reaction mixture, benzyl bromide (21.25 g, 15 mL, 124.0 mmol) was added dropwise. After stirring at room temperature overnight, the reaction was quenched with 1M HCl (150 mL). Benzene was removed under reduced pressure and the residue was taken up in ethyl acetate. The solution was washed with brine, the organic layer was dried over Na_2SO_4, and then the solvent was removed under vacuum to give the crude product. After column chromatography (50% petroleum ether, 50% ethyl acetate), ^{t}Boc-serine benzyl ester **12** was obtained as a white solid (16.90 g, 57.0 mmol, 71%); mp 61-63 °C (lit. mp 69-70 °C); [Found: C, 60.63; H, 7.16; N, 4.64. $C_{15}H_{21}NO_5$ requires C, 61.00; H, 7.17; N, 4.74 %]; ν_{max}/cm^{-1} 3417, 3356 (NH and OH), 1756 (C=O, ester), 1667 (C=O, carbamate), 1523 (amide II); ^1H NMR (300 MHz, $CDCl_3$) δ_H 1.44 (9H, s, $C(CH_3)_3$), 2.24 (1H, br, OH), 3.91 (1H, br d, *J*=11.1 Hz, CH_a-3), 3.98 (1H, br d, *J*=11.1 Hz, CH_b-3), 4.41 (1H, br, CH-2), 5.19 (1H, d, *J*=12.3 Hz, $COOCH_a$), 5.24 (1H, d, *J*=12.3 Hz, $COOCH_b$), 5.44 (1H, br, NH), 7.35-7.37 (5H, m, 5 x CH_{Ar}); ^{13}C NMR (75 MHz, $CDCl_3$) δ_C 27.9 (CH_3, $C(CH_3)_3$), 55.6 (CH, C-2), 63.3 (CH_2), 67.1 (CH_2), 80.1 (quat., $C(CH_3)_3$), 127.8 (2 x CH_{Ar}), 128.1 (CH_{Ar}), 128.3 (2 x CH_{Ar}), 134.9 (quat., C_{Ar}), 153.0 (quat., C=O), 170.3 (quat., C=O); MS (ESI) *m/z* 318.3 (MNa^+).

3.2 ^{t}Boc-β-chloro-L-alanine benzyl ester 13

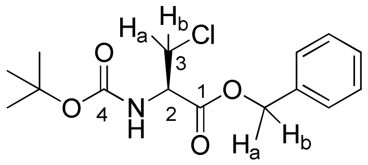

To a solution of ^{t}Boc-serine benzyl ester **12** (15.58 g, 52.8 mmol) in DCM (200 mL), under nitrogen, trichloroacetonitrile (15.16 g, 10.5 mL, 105.0 mmol) was added, followed by 10 minutes of stirring at room temperature. Triphenylphosphine (27.54 g, 105.0 mmol) was dissolved in DCM (150 mL) under nitrogen and this solution was added dropwise to the stirred reaction mixture. After overnight stirring at room temperature, the reaction was quenched with brine (250 mL); after separation, the organic layer was extracted with brine (3x 100 mL). The organic layer was dried over anhydrous sodium sulfate and the solvent was removed under reduced pressure to give the crude product. Purification by column chromatography (70% petroleum ether, 30% ethyl acetate) gave **13** as a white solid (14.94 g, 47.6 mmol, 90%); mp 54-57 °C; [Found: C, 57.53; H, 6.49; N, 4.38. $C_{15}H_{20}ClNO_4$ requires C, 57.42; H, 6.42; N, 4.46 %]; ν_{max}/cm^{-1} 3364 (NH), 1725 (C=O, ester), 1680 (C=O, carbamate), 1518 (amide II); ^{1}H NMR (300 MHz, $CDCl_3$) δ_H 1.45 (9H, s, $C(CH_3)_3$), 3.84 (1H, dd, *J*=3.3 and 11.4 Hz, CH_a-3), 4.00 (1H, dd, *J*=3.3 and 11.4 Hz, CH_b-3), 4.74 (1H, m, CH-2), 5.22 (1H, d, *J*=12.3 Hz, $COOCH_a$), 5.27 (1H, d, *J*=12.3 Hz, $COOCH_b$), 5.43 (1H, d, *J*=7.2 Hz, NH), 7.36 (5H, m, 5 x CH_{Ar}); ^{13}C NMR (75 MHz, $CDCl_3$) δ_C 28.3 (CH_3, $C(CH_3)_3$), 45.5 (CH_2, C-3), 54.6 (CH, C-2), 67.8 ($COOCH_2$), 80.5 (quat., $C(CH_3)_3$), 128.3 (CH), 128.6 (2 x CH), 128.6 (2 x CH), 134.9 (quat., C_{Ar}), 155.0 (quat., C=O), 169.0 (quat., C=O); HRMS (NSI) calcd for $(C_{15}H_{21}NO_4Cl)^+$ 314.1154, found 314.1159.

3.3 β-Chloro-L-alanine benzyl ester hydrobromide 14

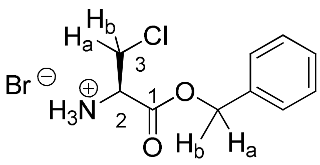

^{t}Boc-β-chloro-L-alanine benzyl ester **13** (1.40 g, 4.46 mmol) was dissolved in a minimum amount of acetic acid (5 ml), then HBr in AcOH (33 w/w %) (5.53 mL, 30.7 mmol of HBr) was added and the reaction mixture was stirred for 10 minutes at room temperature. The reaction was quenched with diethyl ether (200 mL) and the solution was kept in the freezer overnight. A white solid precipitated on standing, which was collected by filtration, and washed with cold diethyl ether to give the product **14** (1.10 g, 3.6 mmol, 81%); mp 131-134 °C; [Found: C, 40.56; H, 4.51; N, 4.68. $C_{10}H_{13}BrClNO_2$ requires C, 40.77; H, 4.45; N, 4.75 %]; ν_{max}/cm^{-1} 2950, 2875, 2846 (br NH_3^+), 1750 (C=O, ester), 1489, 1228, 1208 (C-O); ^{1}H NMR (300 MHz, D_2O) δ_H 4.17 (1H, dd, *J*=3.3 and 12.6 Hz, CH_a-3), 4.31 (1H, dd, *J*=3.3 and 12.6 Hz, CH_b-3), 4.81 (1H, m, CH-2), 5.31 (1H, d, *J*=12.3 Hz, $COOCH_a$), 5.39 (1H, d, *J*=12.3 Hz, $COOCH_b$), 7.55 (5H, m, 5 x CH_{Ar}); ^{13}C NMR (75 MHz, D_2O) δ_C 41.8 (CH_2, C-3), 53.9 (CH, C-2), 69.2 ($COOCH_2$), 128.7 (2 x CH), 128.9 (2 x CH), 129.1 (CH), 134.5 (quat., C_{Ar}), 167.0 (quat., C=O); HRMS (NSI) calcd for $(C_{10}H_{13}{}^{35}ClNO_2)^+$ 214.0629, found 214.0630.

3.4 ^{t}Boc-β-chloro-L-alanine 15 [11,12]

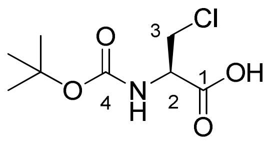

^{t}Boc-β-chloro-L-alanine benzyl ester **13** (1.57 g, 5.0 mmol) was dissolved in methanol (50 mL) and 10 % palladium on charcoal (0.16 g) in ethyl acetate (20 mL) was added. The reaction was stirred under 1.5 bar pressure of H_2 overnight. The catalyst was

removed by filtration through a Celite plug and washed with methanol (200 mL). After removal of the methanol under reduced pressure, the crude product was purified by column chromatography (95% DCM, 5% MeOH) to give **15** as a white solid (0.99 g, 4.4 mmol, 88%); mp 119-123 °C (lit. mp 123-125 °C (13)); ν_{max}/cm^{-1} 3434 (NH), 2975 (OH), 1752 (C=O), 1734 (C=O), 1677, 1521 (amide II), 1370, 1212; ^1H NMR (300 MHz, $CDCl_3$) δ_H1.47 (9H, s, $C(CH_3)_3$), 3.90 (1H, dd, *J*=2.7 and 11.1 Hz, CH_a-3), 4.05 (1H, d, *J*=11.1 Hz, CH_b-3), 4.78 (1H, m, CH-2), 5.47 (1H, d, *J*=6.3 Hz, NH); ^{13}C NMR (75 MHz, $CDCl_3$) δ_C 28.3 (CH_3, $C(CH_3)_3$), 45.2 (CH_2, C-3), 54.3 (CH, C-2), 80.9 (quat., $C(CH_3)_3$), 155.3 (quat., C=O, C-4), 173.2 (quat., C=O, C-1); HRMS (NSI) calcd for $(C_8H_{13}NO_4{}^{35}Cl)^-$ 222.0539, found 222.0541.

3.5 ^{t}Boc-β-chloro-L-alanine pentafluorophenol ester 16

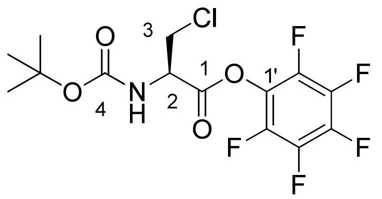

^{t}Boc-β-chloro-L-alanine **15** (1.16 g, 5.2 mmol) and pentafluorophenol (0.95 g, 5.7 mmol) was dissolved in ethyl acetate (25 mL) and cooled in an ice bath. Dicyclohexylcarbodiimide (1.05 g, 5.7 mmol) was added and the solution was stirred for 3 h. The precipitated urea by-product was removed by filtration. The residue was concentrated under reduced pressure and any additional precipitate was removed by filtration. The remaining ethyl acetate was removed by evaporation. The resulting oil was triturated with petroleum ether to give the product **16** as a white solid (3.35 g, 8.6 mmol, 69%) which was collected by filtration; mp 128-131 °C; [Found: C, 43.53; H, 3.49; N, 3.63. $C_{14}H_{13}{}^{35}ClF_5NO_4$ requires C, 43.15; H, 3.36; N, 3.59 %]; ν_{max}/cm^{-1} 3367 (NH),

1780 (C=O), 1681 (C=O), 1518 (amide II); ^{1}H NMR (300 MHz, $CDCl_3$) δ_H 1.48 (9H, s, $C(CH_3)_3$), 3.94 (1H, dd, *J*=3.6 and 11.4 Hz, CH_a-3), 3.96 (1H, dd, *J*=3.6 and 11.4 Hz, CH_b-3), 5.10 (1H, br t, *J*=3.6 Hz, CH-2), 5.46 (1H, d, *J*=7.5 Hz, NH); ^{13}C NMR (75 MHz, $CDCl_3$) δ_C 28.25 (CH_3, $C(CH_3)_3$), 44.78 (CH_2, C-3), 54.55 (CH, C-2), 81.26 (quat., $C(CH_3)_3$), 118.94 (m, C-F_{Ar}), 154.74 (quat., C-1'), 154.79 (quat., C=O, C-4), 166.77 (quat., C=O, C-1); ^{19}F NMR (282 MHz, $CDCl_3$) δ_F -161.67 (2F, t, *J*=19.8 Hz, CF-3' and 5'), -156.73 (1F, t, *J*=23.1 Hz, CF-4'), -151.63 (2F, d, *J*=18.9 Hz, CF-2' and 6'); MS (ESI) *m/z* 388.8 $(M-H)^-$.

3.6 ^{t}Boc-β-chloro-L-alanyl-β-chloro-L-alanine benzyl ester 17

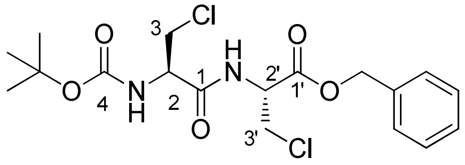

β-Chloro-L-alanine benzyl ester hydrobromide **14** (1.13 g, 3.9 mmol) was dissolved in DMF (30 mL) and added to a solution of ^{t}Boc-β-chloro-L-alanine pentafluorophenol ester **16** (1.50 g, 3.9 mmol) in DCM (10 mL) at 0 °C. To this solution, diisopropylethylamine (0.50 g, 0.66 mL, 3.9 mmol) was added dropwise. The resulting mixture was stirred at room temperature for 2 h, and then heated to 35°C, and the reaction progress was monitored by TLC (80% petroleum ether, 20% ethyl acetate). After the reaction was complete, it was quenched with 1M HCl (50 mL) and the organic layer was washed with water (100 mL) and brine (100 mL). The organic layer was dried over anhydrous sodium sulfate and the solvent was evaporated under vacuum. The crude product was purified by gradient column chromatography (from 80% petroleum ether, 20% ethyl acetate; to 50% petroleum ether, 50% ethyl acetate) to give **17** as a white solid (1.16 g, 2.8 mmol, 70%); mp 89-91 °C; [Found: C, 51.83; H, 5.78; N, 6.78. $C_{18}H_{24}{}^{35}Cl_2N_2O_5$ requires C, 51.56; H,

5.77; N, 6.68 %]; ν_{max}/cm^{-1} 3336 (NH), 3321 (NH), 1739 (C=O), 1655 (m, C=O), 1508 (amide II); ^{1}H NMR (300 MHz, $CDCl_3$) δ_H 1.48 (9H, s, $C(CH_3)_3$), 3.73 (1H, dd, *J*=4.8 and 11.1 Hz, CH), 3.89-4.06 (3H, m, 3 x CH), 4.55 (1H, br, CH), 4.98 (1H, m, CH), 5.24 (2H, m, $COOCH_2$), 5.35 (1H, br, NH), 7.25 (1H, br, NH), 7.37 (5H, br s, 5 x CH_{Ar}); ^{13}C NMR (75 MHz, $CDCl_3$) δ_C 28.24 (CH_3, $C(CH_3)_3$), 33.90 (CH_2Ph), 44.5 (CH), 44.60 (CH_2), 53.61 (CH), 68.13 (CH_2), 81.37 (quat., $C(CH_3)_3$), 128.22 (CH_{Ar}), 128.41 (CH_{Ar}), 128.69 (CH_{Ar}), 134.71 (quat.), 168.19 (2 x quat., C=O), 168.90 (quat., C=O); HRMS (NSI) calcd for $(C_{18}H_{25}N_2O_5{}^{35}Cl)^+$ 419.1135, found 419.1139.

3.7 ^{t}Boc-β-chloro-L-alanyl-β-chloro-L-alanine 18

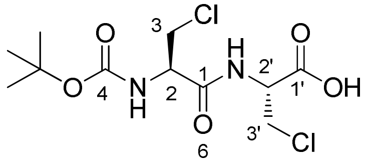

^{t}Boc-β-chloro-L-alanyl-β-chloro-L-alanine benzyl ester **17** (1.00 g, 2.4mmol) was dissolved in methanol (50 mL) and 10 % palladium on charcoal (0.10 g) in ethyl acetate (20 mL) was added. The reaction mixture was stirred under an atmosphere of H_2 (1.8 bar) for 24 h, then was filtered through a Celite plug and washed with methanol (200 mL). The solvents were removed under vacuum and, after purification by column chromatography (95% DCM, 5% MeOH), the product **18** was obtained upon trituration with petroleum ether as a white solid (0.52 g, 1.6 mmol, 66%); mp 72-74 °C; ν_{max}/cm^{-1} 3320 (NH), 2978 (NH), 1724 (C=O), 1665 (C=O), 1530 (amide II); ^{1}H NMR (300 MHz, DMSO-*d6*) δ_H 1.39 (9H, s, $C(CH_3)_3$), 3.67 (1H, dd, *J*=8.4 and 11.1 Hz, CH), 3.79-3.95 (3H, m, CH_2 and CH), 4.36 (1H, br, CH), 4.61-4.67 (1H, m, CH), 7.16 (1H, d, *J*=8.1 Hz, NH), 8.38 (1H, d, *J*=7.5 Hz, NH); HRMS (NSI) calcd for $(C_{11}H_{17}{}^{35}Cl_2N_2O_5)^-$ 327.0520, found 327.0517;

3.8 β-Chloro-L-alanyl-β-chloro-L-alanine 19

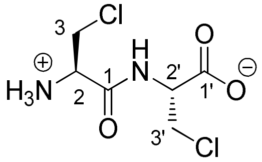

^{t}Boc-β-chloro-L-alanyl-β-chloro-L-alanine **18** (0.73 g, 2.2mmol) was dissolved in a minimum amount of acetic acid (2 mL), then HBr in AcOH (33 w/w %) (4 mL) was added and the reaction mixture stirred for 20 minutes at room temperature. The reaction was quenched with diethyl ether (200 mL) and the solution was placed in the freezer (-30 °C) overnight. The excess diethyl ether was decanted to give an orange oil, which was then triturated 3 times with diethyl ether. The remaining oil was taken up in a minimum amount of methanol (3 mL) and large excess of propylene oxide (150 mL) was added. The precipitate formed was filtered under an inert atmosphere to give the product **19** as a white solid (0.60g, 1.9 mmol, 88%); mp 136-139 °C; ν_{max}/cm^{-1} 3343 (NH), 2971 (NH_3^+), 1671 (C=O), 1624 (amide I), 1521 (amide II), 658 (C-Cl); ^{1}H NMR (300 MHz, D_2O) δ_H 3.89-4.09 (2H, m, CH_2), 4.13-4.25 (2H, m, CH_2), 4.48-4.51 (1H, m, CH), 4.56-4.58 (1H, m, CH); ^{13}C NMR (75 MHz, D_2O) δ_C 42.7 (CH_2), 45.2 (CH_2), 53.9 (CH), 56.4 (CH), 166.2 (quat., C=O), 174.0 (quat., C=O); HRMS (NSI) calcd for $(C_6H_{11}N_2O_3{}^{35}Cl_2)^+$ 229.0141, found 229.0144.

SUPPLEMENTARY REFERENCES

1. Kudzin ZH, Łuczak J. A facile conversion of aminoalkanephosphonic acids into *O*,*O*-dialkyl-*N*-acylaminoalkanephosphonate derivatives. *Synthesis* **1995**, *5*, 509-511.

2. Kudzin ZH, Drabowicz J, Sochacki M, Wiśniewski W. Characterization of 1-aminoalkanephosphonic acids by chemical ionization mass spectrometry. *Phosphorus, Sulfur Silicon Relat. Elem*. **1994**, *92*, 77-93.

3. Yuan C, Xu C, Zhang Y. Enzymatic synthesis of optically active 1- and 2-aminoalkanephosphonates. *Tetrahedron* **2003**, *59*, 6095-6102.

4. Kudzin ZH, Łyżwa P, Łuczak J, Andrijewski G. 1-Aminoalkanephosphonates. Part II. A facile conversion of 1-aminoalkanephosphonic acids into *O,O*-diethyl 1-aminoalkanephosphonates. *Synthesis* **1997**, *1*, 44-46.

5. Tamiaki H, Kiyomori A, Maruyama K. Intramolecular ligation of carbonyl oxygen to central zinc in synthetic oligopeptide-linked zinc porphyrins. *Bull. Chem. Soc. Jpn.* **1994**, *67*, 2478-2486.

6. Takahashi S. Solid-phase syntheses of $Glu_{20}Ala_{20}Phe$ and $Ala_{20}Glu_{20}Phe$ by the step-by-step coupling of dipeptide and tetrapeptide. *Bull. Chem. Soc. Jpn.* **1977**, *50*, 3344-3348.

7. Kametani T, Suzuki Y, Kigasawa K, Hiiragi M, Wakisaka K, Sugi H, Tanigawa K, Fukawa K, Irino O, Saita O, Yamabe S. Studies on the synthesis of chemotherapeutics. Part XIII. Synthesis and biological studies on phosphonopeptides having alkyl-, phenyl-, and heterocyclic substituents. *Heterocycles* **1982**, *18*, 295-319.

8. Huang Y. Ph.D. thesis. Synthesis and evaluation of suicide substrates for the enhanced detection of bacterial pathogens. University of Sunderland, Sunderland, United Kingdom. **2004**.

9. Atherton FR, Hassall CH, Lambert RW. Synthesis and structure-activity relationships of antibacterial phosphonopeptides incorporating (1-

aminoethyl)phosphonic acid and (aminomethyl)phosphonic acid. *J. Med. Chem.* **1986**, *29*, 29-40.

10. Lavielle S, Ling NC, Saltman R, Guillemin RC. Synthesis of a glycotripeptide and a glycosomatostatin containing the 3-*O*-(2-acetamido-2-deoxy-β-D-glucopyranosyl)-L-serine residue. *Carbohydr. Res.* **1981**, *89*, 229-236.

11. Rooseboom M, Vermeulen NPE, Andreadou I, Commandeur JNM. Evaluation of the kinetics of β-elimination reactions of selenocysteine Se-conjugates in human renal cytosol: possible implications for the use as kidney selective prodrugs. *J. Pharm. Experiment. Ther.* **2000**, *294*, 762-769.

12. Miller MJ, Mattingly PG, Morrison MA, Kerwin JF. Synthesis of β-lactams from substituted hydroxamic acids. *J. Am. Chem. Soc.* **1980**, *102*, 7026-7032.
